# Correlates of problematic internet use among undergraduate medical students of Delhi

**DOI:** 10.1186/s12888-021-03529-z

**Published:** 2021-10-15

**Authors:** Deepak Dhamnetiya, Satyavir Singh, Ravi Prakash Jha

**Affiliations:** Department of Community Medicine, Dr Baba Saheb Ambedkar Medical College and Hospital, Sector-6 Rohini, New Delhi, 110085 India

**Keywords:** Undergraduate medical, Internet, Discriminant analysis, Cross-sectional study, Problematic internet use

## Abstract

**Background:**

Globally, due to population diversity, the prevalence of problematic internet use (PIU) varies from 7.3 to 51%. This study aims to assess correlates of problematic internet use among undergraduate medical students of Delhi and derive a model for allocating new subjects among categories of internet users.

**Material and methods:**

A cross-sectional study was conducted on 201 medical-undergraduate students in a medical college of Delhi from April 1st to May 31st, 2019. A semi-structured and pre-tested questionnaire was used to collect demographic information and factors affecting PIU. Dr. Kimberly Young’s Internet Addiction Test (IAT) tool was used to assess PIU. Binary logistic regression has been applied to assess the correlates of PIU, and step-wise discriminant analysis (DA) has been applied to derive a model for allocation of new subjects among categories of internet users. Statistical Package for Social Sciences (Trial version 27.0; SPSS Inc., Chicago, IL) software was used for statistical analysis.

**Result:**

Total 41.3% of the subjects had PIU. Univariate analysis shows that internet use for emotional support, watching adult content, and gambling were significantly associated with PIU; however, in binary logistic regression, chatting, emotional support and watching online adult content were significant risk factors for PIU. The discriminant model correctly classified 66.2% of respondents into average and problematic internet user groups.

**Conclusion:**

We should create awareness among medical students regarding problematic internet use and its potential harms; this could be included in the foundation course of curriculum implementation support program (CISP) for MBBS students.

## Background

The use of the internet has increased dramatically over the past two decades. Recent global internet statistics by global reach show over 803 million people have online access worldwide [1]. The benefits of the internet have been widely researched and include education and research, communication, health-related services, online monetary transactions, trade, buying goods, entertainment, etc. [2]. Researchers found that 73% of college students accessed the internet at least once a day and spent approximately 1.6 to 4.5 h a day online, preferably during the night [2–5]. Globally, due to population diversity, different instruments, cut-off scores used, and different sample characteristics, the prevalence of problematic internet use (PIU) vary from 7.3 to 51% [6–11]; whereas, In India, it ranges from 7.45 to 19.85% among undergraduate medical students [2, 3, 12, 13].

Globally, deteriorating effects of problematic internet use were studied by various researchers; they found that PIU was associated with a wide range of social and psychological problems, like academic failure, poor self-confidence, poor psychological well-being, sleep deprivation, social withdrawal, poor diet, and cardiopulmonary complications [13–15]. The mean scores of the following domains: anxiety, depression, paranoid ideation, and obsessive-compulsive of Symptom Checklist-90-revision (SCL-90-R), were lower in individuals without PIU vis-a-vis PIU [16, 17].

Researchers have suggested various socio-demographic, personal, and internet-related factors are associated with problematic internet use; male gender [4, 18], initial years during the study course, the influence of peers, always logged in status, online interaction with friends, chat, watching porn, online new friendships or relationships, online shopping, average daily time spent on internet and internet access modalities were some of the risk factors for problematic internet use [2, 3, 18, 19]. However, correlates of problematic internet use among undergraduate medical students of North India remain underexplored. Moreover, no research studies have demonstrated a model predicting problematic internet use in a new subject. This study aims to assess the correlates of problematic internet use among undergraduate medical students of Delhi and derive a model for allocating new subjects among categories of internet users.

## Material and methods

A cross-sectional study was conducted on 201 undergraduate students in a medical college of Delhi, India, from April 1st to May 31st, 2019. **Inclusion criteria:** All the undergraduate students studying in a medical college in Delhi and using the internet at least for the last six months were selected for the study. **Exclusion criteria:** those who do not give consent. **Sample size calculation:** A total of 300 students were enrolled in the medical college, 100 students in each batch. The sample size was calculated by taking prevalence of PIU as 50% at a level of 95% significance, 5% precision, and population size as 300.

*n* = [Np (1-p)]/ [(d^2^/Z^2^_1-α/2_*(N-1) + p*(1-p)].

Where, *n* = sample size, *N* = population size, *p* = prevalence, *d* = precision.

The required minimum sample size comes up to be 169; Considering a non-response rate of 10%, the final minimum sample size was 188. We have studied and analyzed data from 201 students.

### Questionnaire design and validation

The pre-testing of semi-structured questionnaire was done on 20 undergraduate students. Some questions were modified after pre-testing. The language of some questions was improved for better clarity to the respondent. Reasons for internet use questions were changed from open-ended to 15 close-ended questions (as per responses received) with dichotomous responses “Yes” or “No”, and one open-ended question were added for any other reason of internet use. This pre-tested questionnaire was used to collect information regarding age, gender, socio-economic status, place of residence, year of admission, ownership of gadget (computer, laptop, mobile, tablet), and questions related to internet use; preferred place of internet access (home, cybercafé, or others), for how long have you used the internet? On average, how much time per week do you spend on the internet? On average, how much money per month do you spend on the internet. Questions related to reasons for internet use; why do you use the internet (for communicating with friends and family, required for course work/ assignments, research on new developments/ in areas of interest, browsing, news updates, recreation or relaxation, meeting new people, chatting with others to share interests/ ideas or fantasies, time pass, emotional support, job search, gambling, adult-only content, games, and shopping, etc.). on an average, how much time per week do you spend on the internet sites like WhatsApp, online movies, online shopping, search tool (Google/Bing, etc.), adult content site, email, torrent download, duration of internet use, money spent on the internet per month, Snapchat, Twitter, YouTube, Facebook, newsgroup, gaming sites, spiritual content, music/songs, and Instagram.

We have used Dr. Kimberly Young’s internet addiction test (IAT) scale to assess PIU [20]. The IAT is a 20-item that measures the severity of self-reported compulsive use of the internet. Each item is rated on a 6-point Likert scale ranging from 0 to 5; 0 = Not Applicable, 1 = Rarely, 2 = Occasionally, 3 = Frequently, 4 = Often, 5 = Always. The marking for this questionnaire ranges from 0 to 100; the higher the score range, the greater the level of addiction. Subjects with scores < 50 were categorized as “average internet users,” and those with scores ≥50 were categorized as “problematic internet users. The Cronbach’s α computed from the studies was 0.889 [95% confidence interval (CI) 0.884–0.895]. The standard deviation of the alpha was low, at 0.049 [21]. In the present study, we have found high internal consistency, with an alpha coefficient of 0.889 (CI 0.867–0.911).

### Data collection

We have stratified undergraduate students according to the year of admission and enrolled at least 50 students from each stratum. We have fixed the criteria that at least 60% of students should be present in each class. Out of the present students, 90% were chosen randomly using computer-generated random numbers by giving a serial number to the present students. Single attempts were made to collect data from each admission year student. A semi-structured and pre-tested questionnaire was distributed among randomly selected students, and they were asked to fill the questionnaire once**.** The researchers had explained the purpose and objectives of the study to the participants. Participants were informed that participation is voluntary and it will not affect their grades.

### Data management and statistical analysis

Confidentiality of all the data was ensured by keeping the responses anonymous**.** Moreover, the collected data was stored under secure settings**.** Data was recorded in MS Excel, and the trial version of the statistical package for social sciences (version 27.0; SPSS Inc., Chicago, IL) software was used for statistical analysis. No missing data was encountered. Categorical data were described as frequencies and percentages. The unadjusted and adjusted odds ratio was calculated by applying binary logistic regression to assess the correlates of PIU. A *p*-value of less than 0.05 is considered significant for all analyses. Step-wise discriminant analysis (DA) has been applied to derive a model for allocating new subjects among categories of internet users.

The analysis creates a discriminant function, a linear combination of the weightings and scores on these variables. The maximum number of functions is either the number of predictors or the number of groups minus one, whichever of these two values is smaller [22]. The discriminant analysis involves determining a linear equation like a regression that will predict which group the case belongs to. The form of the equation or function is:

Z_jk_ = a + W_1_X_1k_ + W_2_X_2k_ + ... + W_n_X_nk_.

Where:

Z_jk_ = Discriminant Z score of discriminant function j for object k.

a = Intercept.

W_i_ = Discriminant coefficient for the Independent variable i.

X_ik_ = Independent variable i for object k.

*n* = number of predictor variables.

## Results

In the present study, we have analyzed data of 201 subjects; the majority of the study subjects were ≥ 20 years. Approximately 2/3rd of the subjects was males & 96% followed the Hindu religion. Majority of the subjects (76.1%) belongs to nuclear family and upper or upper-middle SES (84.6%). Approximately 3/4th of the study subjects (74.1%) had permanent residence in Delhi, and more than half of the subjects (55.7%) stayed in the hostel. 36.8% of the study subjects were in the third year, 36.3% in the second year, and 26.9% in the first year (Table 1).

**Table 1:** Distribution of study subjects according to socio-demographic characteristics (*N* = 201)

Variable	Frequency (%)
Age	
< 20 years	59 (29.4)
≥ 20 years	142 (70.6)
Gender	
Male	132 (65.7)
Female	69 (34.3)
Religion	
Hindu	193 (96)
Muslim or Sikh	8 (4)
Type of family	
Nuclear	153 (76.1)
Joint	48 (23.9)
Socio-Economic Status	
Upper & Upper Middle	170 (84.6)
Lower & Lower Middle	31 (15.4)
Permanent residence	
Delhi	149 (74.1)
Non-Delhi	52 (25.9)
Hostel accommodation status	
Hosteller	112 (55.7)
Non-Hosteller	89 (44.3)
Admission year	
2018 (First year)	54 (26.9)
2017 (Second year)	73 (36.3)
2016 (Third year)	74 (36.8)

Two third of the study subjects were started using the internet during their early adolescent period. Most of the study subjects (60.7%) used the internet for 6–10 years, and only 11.4% of study subjects used the internet for more than ten years. Almost all the study subjects had smartphones (99%), 51.7% had laptops, 31.3% had computers, and 24.4% had tablets. Only 15.4% of subjects had all the above electronic gadgets. Almost all the subjects (99%) preferred smartphones for internet access. The majority of subjects access the internet daily, more than half (51.7%) of the study subjects preferred night time to access the internet, and only 9% of subjects preferred morning time to access the internet. The majority of subjects (72.1%) used the internet less than 5 h a day, and only 6% used the internet for more than 10 h a day. More than half (50.7%) of the study subjects had spent less than INR 150 per month on the internet, and only 10% of study subjects had spent more than INR 500 per month on the internet. 60.7% of study subjects were permanently logged in, and 41.3% had PIU (Table 2).

**Table 2:** Pattern of Internet use among study subjects (*N* = 201)

Variable	Frequency	Percentage
Age at first internet use		
5–10 years	32	15.9
11–15 years	133	66.2
16–20 years	36	17.9
Duration of internet use		
1–5 years	56	27.9
6–10 years	122	60.7
> 10 years	23	11.4
Ownership of electronic gadget with internet access^*^		
Smartphone	199	99
Laptop	104	51.7
Computer	63	31.3
Tablet	49	24.4
All	31	15.4
The most common mode of internet access		
Smartphone	199	99
Computer	2	1
Internet use per week		
7 days	192	95.5
2–6 days	9	4.5
Preferred time to use internet		
Day (6 am to 5 pm)	18	9
Evening (5 pm–10 pm)	79	39.3
Night (10 pm-5 am)	104	51.7
Internet use per day		
≤ 5 Hours	145	72.1
6–10 Hours	44	21.9
> 10 Hours	12	6.0
Money spent on the internet per month		
INR 1–150	102	50.7
INR151–300	48	23.9
INR 301–500	31	15.4
INR > 500	20	10
Log in status		
Permanently login	122	60.7
On and off	79	39.3
Problematic internet use		
Yes	83	41.3
No	118	58.3

* Multiple response

**Fig. 1:**
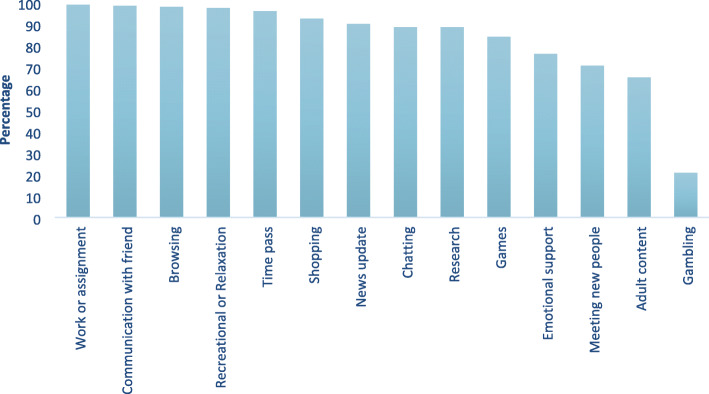
Reasons of internet use among study subjects (*N* = 201)

Common reasons for internet usage found to be work or assignment, communication with friends, browsing, recreational or relaxation purposes, time pass, shopping, and news update, i.e., 99, 98.5, 98, 97.5, 96, 92.5, and 90% respectively. About 21% of the subjects used the internet for gambling (Fig. 1). Univariate analysis shows that internet use for emotional support, watching adult content, and gambling were significantly associated with PIU; however, in binary logistic regression, chatting, emotional support and watching online adult content were found to be significant risk factors for PIU (Table 3).

**Table 3:** Association of selected risk factors with Problematic Internet Use (*N* = 201)

Variable	Average Internet users (118)	Problematic Internet users (83)	Unadjusted OR (95% CI)	Adjusted OR (95%CI)
Gender (Male)	76	56	1.15 (0.63–2.08)	0.68 (0.32–1.46)
Permanent residence (Delhi)	93	59	1.51 (0.79–2.89)	0.49 (0.21–1.10)
Hostel accommodation status (Yes)	67	45	0.90 (0.51–1.59)	0.63 (0.31–1.31)
Admission year (2016 & 2017)	85	62	1.15 (0.61–2.17)	1.352 (0.65–2.80)
Age at first internet use (> 10 Years)	18	14	1.13 (0.53–2.42)	1.34 (0.55–3.28)
Preferred time of day for internet access (Evening or Night)	105	78	1.931 (0.66–5.64)	2.62 (0.78–8.86)
Work or assignment (Yes)	117	82	1.4 (0.09–23.14)	1.35 (0.06–30.16)
Communication with friend (Yes)	117	81	2.90 (0.26–32.39)	1.31 (0.08–20.98)
Browsing (Yes)	116	81	1.43 (0.20–10.38)	1.77 (0.19–16.08)
Recreational or Relaxation (Yes)	115	81	0.95 (0.16–5.79)	0.24 (0.02–3.38)
Wasting time (Yes)	112	81	0.46 (0.09–2.34)	1.14 (0.17–7.50)
Shopping (Yes)	110	76	1.27 (0.44–3.64)	1.95 (0.55–6.91)
News update (Yes)	107	74	1.18 (0.47–3.00)	0.96 (0.31,2.99)
Chatting (Yes)	106	72	1.35 (0.57–3.23)	3.44 (1.02–11.53) *
Research (Yes)	106	72	1.35 (0.565–3.23)	2.04 (0.62–6.66)
Games (Yes)	98	71	0.83 (0.38–1.80)	0.77 (0.30–1.99)
Emotional support (Yes)	81	72	0.33 (0.16–0.70) *	0.28 (0.12–0.69) *
Meeting new people (Yes)	79	63	0.64 (0.34–1.21)	0.72 (0.30–1.70)
Adult content (Yes)	69	62	0.48 (0.26–0.88) *	0.37 (0.16–0.81) *
Gambling (Yes)	19	23	0.50 (0.25–1.00) *	0.65 (0.29–1.46)

* *p* < 0.05

In Table 4, The test of equality of group means has been performed to measure each independent variable’s potential before the model is created. Each test displays the results of a one-way ANOVA for the independent variable using the grouping variable, i.e., Internet Users as the factor. If the *p*-value value is greater than 0.05, the variable probably does not contribute to the model. Wilks’ lambda is another measure of a variable’s potential. Smaller values indicate the variable is better at discriminating between groups. We have found strong statistical evidence of significant differences between means of average internet users and problematic internet users for only seven variables naming email time (in min), shopping time (in min), YouTube time (in min), WhatsApp time (in min), movie time (in min), download time (in min) and educational use time (in min). In contrast, insignificant variables are not suitable to discriminate between average internet users and problematic internet users (Table 4).

**Table 4:** Test of equality of group means of studied variables among categories of internet users

Variables	Wilks Lambda	F value	p-value
Age	0.995	1.096	0.296
Family Income (INR)	0.987	2.610	0.108
Amount Spent on Internet (INR)	0.997	0.570	0.451
Email time (in min)	0.972	5.651	0.018*
Tool time (in min)	0.985	3.042	0.083
Newsgroup time (in min)	0.988	2.432	0.120
Game site time (in min)	1.000	0.006	0.938
Shopping time (in min)	0.972	5.698	0.018*
You Tube time (in min)	0.977	4.607	0.033*
Music time (in min)	0.991	1.881	0.172
Facebook time (in min)	0.993	1.430	0.233
WhatsApp time (in min)	0.952	10.099	0.002*
Twitter time (in min)	0.989	2.304	0.131
Instagram time (in min)	0.995	1.001	0.318
Snapchat time (in min)	0.990	2.043	0.155
Movie time (in min)	0.971	5.883	0.016*
Download time (in min)	0.969	6.372	0.012*
Educational use time (in min)	0.972	5.733	0.018*
Spiritual time (in min)	0.992	1.664	0.199
Adult site time (in min)	0.986	2.854	0.093

* *p*-value< 0.05

The step-wise discriminant analysis method has been applied for selecting the “best” variables to use in the model. The step-wise method starts with a model that doesn’t include any of the independent variables. At each step, the predictor with the largest F value to Enter a value that exceeds the entry criteria 3.84 is added to the model. At the last step, the variables left out of the analysis all have F to Enter values smaller than 3.84, so no more are added. So, the final selected variables in the model having F to enter value > 3.84 are family income, email time, and WhatsApp time. The F value for a variable indicates its statistical significance in the discrimination between groups, i.e., it is a measure of the extent to which a variable makes a unique contribution to the prediction of a group membership.

The equation of the model while considering the variables selected by applying step-wise discriminant analysis is as follows:

D = (0.000* family income) + (0.0076* email time) + (0.001*WhatsApp time)– 0.294.

**Fig. 2:**
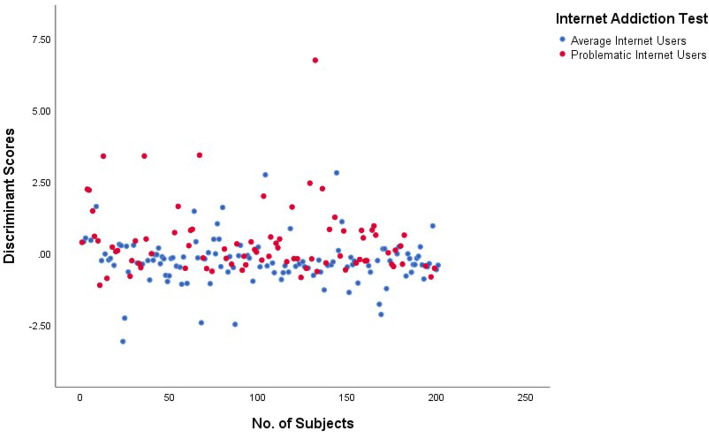
Scatter plot of discriminant scores of each subject for the model

We can calculate the discriminant scores by putting the values of these three variables in the above discriminant equation, by comparing this discriminant score with the cut-off value (Fig. 2), we can predict the allocation of subjects in average internet users or problematic internet users’ group.

Table 5 shows that 66.2% of respondents were correctly classified into average internet users and problematic internet users’ groups. This model correctly predicts 92.4% of subjects with average internet use.

**Table 5:** Classification result table of the proposed model by applying Step-wise discriminant analysis model (*N* = 201)

Original classification	Predicted Group Membership		Total
	Average Internet Users (%)	Problematic Internet Users (%)	
Average Internet Users	109 (92.4)	9 (7.6)	118 (58.7)
Problematic Internet Users	59 (71.1)	24 (28.9)	83 (41.3)

Figure 2 shows the discriminant scores of 201 subjects. The centroid value for average internet users is − 0.278, whereas for problematic internet users is 0.395.

The general formula for the calculation of cut off value is given by

$$ {Z}_{cs}=\frac{N_A{Z}_B+{N}_B{Z}_A}{N_A+{N}_B} $$

Where,

Z_CS_ = Optimal cut-off value between groups A and B.

N_A_ = number of observations in group A.

N_B_ = number of observations in group B.

Z_A_ = Centroid for group A.

Z_B_ = Centroid for group B.

So, in this case, the cut-off score will be 0.118. Hence the cut-off values above 0.118 are classified as problematic internet users, and below 0.118 are classified as average internet users.

## Discussion

In our study, we have found 41.3% of the subject had PIU; this has been corroborated with a study conducted on medical students by Pramanik et al. [9]; whereas in some other studies, the PIU ranges from 5.8–30% [3, 7, 8, 12, 23–25]; however, a study conducted by Sayyah et al. [11] found a high prevalence of PIU (51%). The high magnitude of PIU in our study may be due to the demographic profile of study subjects as a majority (84.6%) belongs to upper or upper-middle SES and increased penetration of internet in metro cities like Delhi. We have found no significant association between gender and PIU; similar results were found in a study conducted by other researchers [26, 27], whereas most of the studies showed that male gender was significantly associated with PIU [2, 3, 6, 7, 23, 25, 28, 29] However, previous studies found that females were significantly associated with PIU [24, 30]. No significant association of gender with PIU in our study may be due to the fact that good accessibility to internet among male and female medical students. In this study, no association was seen between hostel accommodation and PIU; the same results were found by Salehi et al. [6] and Ghamari et al. [30]; whereas PIU was significantly higher in hostellers vis-a-vis non-hostellers in studies conducted by Chaudhari et al. [2] and Anand et al. [29]. We have found no association of PIU with the year of study, and the same result was found by Chaudhari et al. [2]. In contrast, Krishnamurthy et al. [12] and Asiri et al. [31] were found that students in first or second professional years had significant higher PIU as compare to third- and fourth-year students; however, Sayyah et al. [11] found that PIU was significantly higher in senior students as compared to junior students. The reason of no association of PIU with the year of study in present study may be due to sharing of similar psychological and environment factors among medical students of all the professional years. We have found no significant association of PIU with age at first use of the internet. In contrast, some authors found age at first use of the internet was significantly lower in students with PIU [2, 28]. We have found no association of PIU with a preferred time of internet use; this has been corroborated with a study conducted by Salehi et al. [6]; whereas Gedam et al. [3] found PIU was significantly higher in students whose preferred time of internet access was evening or night vis-a-vis morning or afternoon.

Our study shows that internet usage for emotional support, watching adult content, gambling and chatting was a statistically significant risk factor for PIU; this had been corroborated with studies conducted by several researchers [2, 7, 12, 23, 32]. We have found that internet usage for work or assignment, communication with a friend, browsing, recreational or relaxation, wasting time, shopping, news update, research, games, and meeting a new person on social media were not significantly associated with PIU, identical results were found in various studies conducted in India and other countries as well [3, 6, 7, 28]. In contrast, Salehi et al. [6] found that communication with friends was significantly associated with PIU & in a study conducted by Krishnamurthy et al. [12], internet use for work and making new friends on social media was significantly associated with PIU. A study conducted by Mazhari [7] found that the internet used for shopping was significantly associated with PIU. To the best of our knowledge, the present study is the first that discriminate a new subject in the average and problematic internet user groups. The model derived from step-wise DA suggests that family income, email time and WhatsApp time discriminate 66.2% of the subjects correctly into average and problematic internet user groups. WhatsApp is one of the commonest social networking applications used to share text massages, videos, photos, and work-related information which leads to its excessive use in everyday life [33]. Driving factor for the spread of WhatsApp use is its convenience; people may access massages and reply from anywhere anytime. Income is one of the factors that directly correlates with internet use. High income leads to more use of internet [34]. There is increase in the use of email for academic work, assignment and research related activity among medical students. These discriminators can be used to determine the PIU among undergraduate medical students.

Our study has several limitations; First, it’s a single-centre study, so multi-centre studies are warranted and explore the differences in areas, specialties, and grades. Second, being a cross-sectional study, we could not establish a cause-and-effect relationship; a longitudinal study would be more informative. Third, this study is subject to some recall bias. Fourth, the use of self-report for measuring time spent on a range of devices and activities by a person is likely to be biased.

## Conclusion

Our study reported high PIU among undergraduate medical students. Internet usage for emotional support, watching online adult content, and chatting was significantly associated with PIU. We should create awareness among medical students regarding PIU and its potential harms; this could be included in the foundation course of the curriculum implementation support program (CISP) for MBBS students. The initiative should be taken to create ample opportunities for students to involve in extracurricular activities and interact with friends. There should be a provision of counsellors for emotional and mental support of medical students as they are overburden with studies and long posting schedules.

## Data Availability

The study datasets are available from the corresponding author on reasonable request.

## Abbreviations

CI: Confidence Interval
CISP: Curriculum Implementation Support Program
DA: Discriminant Analysis
IAT: Internet Addiction Test
INR: Indian National Rupee
OR: Odds Ratio
PIU: Problematic Internet Use
SES: Socio-Economic Status
